# Structural and Functional Aspects of Foamy Virus Protease-Reverse Transcriptase

**DOI:** 10.3390/v11070598

**Published:** 2019-07-02

**Authors:** Birgitta M. Wöhrl

**Affiliations:** Lehrstuhl Biopolymere, Universität Bayreuth, D-95440 Bayreuth, Germany; birgitta.woehrl@uni-bayreuth.de

**Keywords:** foamy virus, protease, reverse transcriptase, RNase H, reverse transcription, antiviral drugs, resistance

## Abstract

Reverse transcription describes the process of the transformation of single-stranded RNA into double-stranded DNA via an RNA/DNA duplex intermediate, and is catalyzed by the viral enzyme reverse transcriptase (RT). This event is a pivotal step in the life cycle of all retroviruses. In contrast to orthoretroviruses, the domain structure of the mature RT of foamy viruses is different, i.e., it harbors the protease (PR) domain at its N-terminus, thus being a PR-RT. This structural feature has consequences on PR activation, since the enzyme is monomeric in solution and retroviral PRs are only active as dimers. This review focuses on the structural and functional aspects of simian and prototype foamy virus reverse transcription and reverse transcriptase, as well as special features of reverse transcription that deviate from orthoretroviral processes, e.g., PR activation.

## 1. General Features of Foamy Virus Replication

Foamy viruses (FVs) are retroviruses that—based on several differences in their molecular properties—are gathered in the subfamily of *Spumaretrovirinae*, whereas all other retroviruses are members of the subfamily *Orthoretrovirinae* [1]. The latter include well-characterized retroviruses such as human immunodeficiency virus (HIV), murine leukaemia virus (MLV), or Rous sarcoma virus (RSV) [2]. FVs are endemic in various mammalian hosts, including cats, horses and non-human primates, but not humans. The so-called prototype foamy virus (PFV) was first isolated from a human nasopharyngeal cell line [3]. Sequence comparisons with a simian FV revealed that it originally was derived from a chimpanzee [4].

**Figure 1:**
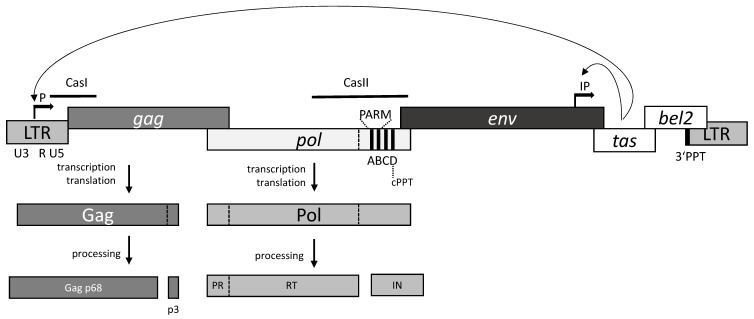
Overview of the foamy virus (FV) genome organisation. The proviral DNA genome is shown. The *gag, pol,* and *env* genes are depicted as boxes. The flanking long terminal repeats (LTRs) comprise the U3, R, and U5 regions, as indicated underneath the 5′ LTR. Transcription starts at the promoter upstream of the R region in the 5′ LTR and at the internal promoter (P and IP, respectively), which are depicted as rectangular arrows. The transactivator protein Tas activates both promoters, as indicated by the arrows. *bel2* encodes the Bet protein. The locations of the Cas sequences, PARM, the purine rich elements A–D, as well as the cPPT and 3′ PPT are illustrated. Only the gene products Gag and Pol, which are processed by the viral PR, are shown.

FVs are complex retroviruses, i.e., they contain accessory genes. Similar to orthoretroviruses, their genomes contain the genes *gag*, *pol,* and *env* (Figure 1). However, in contrast to orthoretroviruses such as human immunodeficiency virus (HIV), the Pol protein is expressed from a separate mRNA and translated from its own AUG start codon; thus, no Gag–Pol fusion protein is produced [5,6,7].

In the proviral genome, the viral genes are flanked by long terminal repeats (LTRs). The 5′ LTR harbors the viral promoter, which controls transcription of the *gag*, *pol*, and *env* mRNAs. However, additionally, FVs possess an internal promoter (IP) near the 3′ end of the *env* gene, which is responsible for transcription of the accessory proteins Bet and Tas [8,9,10,11] (Figure 1). Tas activates transcription from the 5′ LTR and enhances transcription from the IP [12]. The Bet protein appears to be important for efficient virus replication [13], and interacts with the cellular proteins of the APOBEC family, which function as antiretroviral restriction factors [14,15,16,17,18].

Another interesting feature of FVs is the processing of the Gag protein. Whereas in orthoretroviruses, Gag is cleaved into matrix (MA), nucleocapsid (NC), and capsid (CA) proteins, the only cleavage in the 71-kDa Gag of FV occurs near the C-terminus, resulting in a 68-kDa Gag and a ca. 3-kDa peptide (Figure 1). The cleavage of Gag by the viral protease (PR) was shown to be essential for infectivity [19,20,21]. The wild-type virus contains a mixture of Gag p71/p68 proteins at a ratio of ca. 1:4 [22]. Inactivation of the Gag p68/p3 cleavage site inhibits reverse transcription at the first template switch. However, p3 itself is not required for infectivity [20,23,24,25,26].

## 2. The Pol Protein

Conventional retroviruses express *pol* as a Gag–Pol fusion protein by a rare frameshift event or nonsense codon suppression mechanism. In FVs, Pol is generated from a spliced mRNA independently from Gag [5,6,7,27,28,29]. It contains the genes for the PR, polymerase, and RNase H domains, forming the reverse transcriptase (RT) as well as the integrase domain (IN) (Figure 1). The FV Pol protein undergoes only limited proteolysis. A single cleavage between the RNase H and IN domains is carried out, resulting in two mature viral enzymes IN and a PR–RT fusion protein [30,31]. This is in contrast to HIV and other orthoretroviruses, in which Pol is cleaved by the viral PR into three separate proteins, PR, RT and IN (reviewed in [32]).

The existence of a separate Pol poses interesting questions regarding its encapsidation into the FV capsid. It has been suggested that only very few Pol molecules are encapsidated [33,34]. Various studies identified two *cis*-acting sequences (Cas) in the FV pregenomic RNA: CasI and CasII (Figure 1), which are essential and sufficient for the transfer of FV vectors, indicating an important role in virus assembly [35,36,37,38]. CasI spans from the 5‘ leader sequence into the 5‘ *gag* region of the pregenomic RNA of PFV (nucleotides 1–645). CasII is situated in the 3′ region of *pol* (nucleotides 3869–5884) [39]. Within the Cas regions, so-called Pol encapsidation sequences (PES) have been detected that are required to incorporate the full-length Pol protein into the FV capsid. These PES regions range from nucleotides 314 to 354 in CasI and nucleotides 4881 to 5884 in CasII. The deletion of either PES resulted in a significant reduction of Pol uptake into the virus particles [39].

Furthermore, FV Gag binds to pregenomic RNA, and its C-terminus contains determinants that are also important for Pol encapsidation [24,37,39,40]. These results indicate that the pregenomic RNA functions as a bridging molecule between Gag and Pol precursors, and that an interplay of protein–protein as well as protein–RNA interactions is important for correct virus assembly.

## 3. Reverse Transcription

Reverse transcription—the reverse flow of genetic information from RNA to DNA—is pivotal in the replication cycle of all retroviruses. In the year 1970, the enzyme reverse transcriptase (RT), which catalyses this process, was identified [41,42]. Retroviral RTs exhibit two enzymatic activities that are required to synthesize double-stranded DNA from a single-stranded RNA template: (1) a DNA polymerase activity that can use both DNA and RNA as a template, and (2) an RNase H endonuclease activity that hydrolyzes the RNA strand in an RNA/DNA intermediate. Without the RNase H activity, reverse transcription cannot take place, since RNA degradation is absolutely required for synthesis of the second DNA strand. Misleadingly, the polymerase domain alone is often called the RT domain.

Although the principal order of events is similar in orthoretroviruses and spumaretroviruses, FV reverse transcription takes place late in the replication cycle, i.e., shortly before the virus leaves the cell, whereas conventional retroviruses reverse transcribe their genomic immediately after entering the cell. The pregenomic single-stranded RNA is packaged during virus assembly and reverse transcribed into double-stranded DNA before budding. Thus, the virions of FVs contain mainly double-stranded DNA, which is the functional genome when the virus infects the cell. The packaged pregenomic RNA is diploid. A dimerization signal has been identified at the 5′ end of the RNA [43,44].

Experiments with the RT inhibitor 3′ azido-3′deoxythymidine (AZT) revealed that reverse transcription is largely complete before the infection of new cells [5,45,46]. However, the results of Delelis and Zamborlini suggested a biphasic DNA synthesis with an additional early reverse transcription event, which might optimize genome replication [47,48]. In contrast, in conventional retroviruses such as HIV-1 and MLV, only a very small amount of DNA consisting only of early reverse transcription products—but no full-length DNA—could be detected in virions [49].

In some aspects, FVs resemble hepatitis B virus (HBV). Similar to the FV Gag, the HBV viral structural core protein is not cleaved in virions, and contains Arg-rich regions that interact with RNA in the early stages of reverse transcription and with DNA during encapsidation and in the mature particle [50,51]. In HBV, long reverse transcription products are synthesized by the reverse transcriptase, which is called the P protein. Interactions between the viral pregenomic RNA, the P protein, and the core protein are necessary for particle assembly. In extracellular HBV particles, a partially double-stranded (gapped) circular DNA molecule is present instead of RNA, indicating that reverse transcription takes place during and after particle formation, but before the virus enters a new cell [52,53].

Several groups have investigated the effect of PFV mutants expressing *gag* and *pol* as a Gag–Pol fusion protein [54,55,56,57]. The co-expression of Gag with Gag–Pol resulted in a molar ratio of 20:1 in virus particles, which is similar to orthoretroviruses. However, larger variations in the Gag:Pol ratio than in orthoretroviruses are tolerated. Furthermore, virus titers similar to that of the wild type could be achieved as long as a proteolytic cleavage took place between Gag and Pol [54,56]. If the constructs did not allow for removal of the p3 Gag peptide from Pol, particle release resembled that of the wild type, but infectivity was reduced [56]. Reverse transcription with the Gag–Pol mutant virus was also found to be a late event in the replication cycle. However, under AZT treatment, a ca. fivefold drop in virus titer was determined for both wild-type and mutant viruses, implying that early DNA synthesis might also be required [54,57].

Similar to all retroviruses, FV reverse transcription starts at the so-called primer binding site (PBS) close to the 5′ RU5 region of the pregenomic RNA (Figure 1). PFV uses a tRNA^Lys1,2^ primer annealed to the PBS for minus-strand DNA synthesis [30]. Synthesis of the plus-strand DNA is initiated at the 3′ polypurine tract (PPT), which is located upstream of the 3′ U3R region. Additionally, FVs harbor a second so-called central PPT (cPPT), which is located in the CasII region of the *pol* open reading frame. Within CasII, four purine rich sequences (elements A–D) are present. However, only the D element is 100% identical to the 3′ PPT, and thus is likely to constitute the actual cPPT (Figure 1). It is highly conserved in all FV species [58,59]. The C element is required for the regulation of gene expression, and appears to be relevant in *cis* to achieve a sufficient amount of Gag protein. It has been shown recently that it regulates splicing by suppressing the branch point recognition of the strongest *env* splice acceptor. Thus, it plays an essential role in the formation of unspliced *gag* and singly spliced *pol* transcripts [39,58,59,60,61]. A and B elements play a role in Pol encapsidation and moreover in PR activation (see below) [59]. Similar to lentiviruses such as HIV, the cPPT of FVs is used as a second initiation site for plus-strand DNA synthesis. In HIV, a so-called central flap region with overlapping single-stranded DNAs is created during reverse transcription. The flap ensures efficient replication in non-dividing cells [62]. However, FVs are not able to establish productive infection in resting cells [63]. Instead of creating a flap, the cPPT is degraded to produce a single-stranded gap region in the double-stranded unintegrated linear PFV DNA [58,59,60]. The length of the PFV gap varies from 144 to 731 nucleotides with the start and terminal nucleotides being located on either side of the cPPT D element. Mutations in the FV cPPT, which retain the IN amino acid sequence, result in the reduction of the virus titer, indicating the important role of the cPPT in virus replication [59,64]

## 4. Foamy Virus PR-RT

### 4.1. Domain organization.

**Figure 2:**
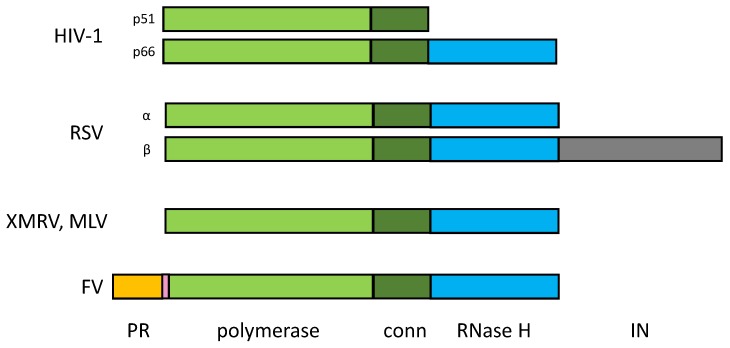
Domain organisation of retroviral RTs. Human immunodeficiency virus (HIV-1) reverse transcriptase (RT) is a heterodimer with a 66-kDa and a 51-kDa subunit. The sequence of the N-terminal region of the two subunits is identical, and comprises the polymerase domain and the connection subdomain, which are highlighted in light and dark green, respectively. The RNase H domain (blue) is located at the C-terminus of the larger subunit. The Rous sarcoma virus (RSV) RT is also heterodimeric. The larger β subunit (95 kDa) carries, in addition to the polymerase, connection, and the RNase H (sub-)domains of the small α subunit (63 kDa), the IN domain (grey). Xenotropic murine leukaemia virus-related virus (XMRV) and murine leukemia virus (MLV) RTs are monomeric enzymes (75 kDa). In addition, the mature monomeric FV enzyme (86 kDa) harbors the PR domain. The stretch ranging from amino acids (aas) 102–143 between the C-terminal end of the PR domain, and the start of the RT domain is highlighted in pink.

Although the RTs of retroviruses all fulfill the same essential function, i.e., the formation of double-stranded DNA from a single-stranded RNA template, their domain organization is different (Figure 2). HIV RT is a heterodimeric enzyme in which only the larger p66 subunit harbors the polymerase active site and carries the RNase H domain located at the C-terminus. The p51 domain is homologous to the N-terminus of p66, but lacks the RNase H domain, which is cleaved off by the viral PR (Figure 2). Due to the different conformations of p51, no polymerase active site can be formed [65,66]. The RT of RSV is also heterodimeric, consisting of a 63-kDa α subunit and a 95-kDa β subunit, although the respective homodimers can also be isolated from virus particles [67,68]. In addition to the polymerase domain, the connection subdomain and the RNase H domain, the β subunit harbors the IN domain. The active sites of both the polymerase and RNase H are located in the α subunit [69].

The RT of Moloney MLV (MoMLV) and the closely related xenotropic murine leukaemia virus-related virus (XMRV) are monomeric enzymes. The RNase H domain is connected to the polymerase domain via a flexible linker and thus is quite mobile, but becomes ordered in the presence of substrate [70,71]. The mature RT from FVs is actually a PR-RT fusion protein harboring the PR domain at its N-terminus [72,73]. Nevertheless, FV PR-RTs resemble MLV RT in their structural organization and in some biochemical and biophysical properties, but differ from HIV RT. However, since the overall amino acid similarity of FV RTs to MoMLV or XMRV RT is less than 25%, and the PR domain is an integral part of the mature FV enzyme, subdomain assignments cannot be easily obtained from sequence comparisons. Size exclusion chromatography with purified PR-RTs of SFV from macaques (SFVmac) and PFV showed that they are monomers in solution [74,75]. They exhibit polymerase as well as RNase H activities [33,74,76]. Furthermore, similarly to MLV, the isolated RNase H domain is active, but loses specificity (see below) [77,78,79].

Purified recombinant FV PR-RT monomers do not exhibit PR activity, nor does the separate PR domain. The PR activity of the full-length PR-RT and the separate PR domain can be induced by unphysiological high NaCl concentrations of 3–4 M (see below) [74,80,81]. Unfortunately, no crystal structure of a full-length PR-RT enzyme is available so far. However, the NMR solution structures of isolated FV PR and RNase H domains exist, which give insight into the functions of the FV PR-RT enzyme and its domains [75,77,78,80]. Amino acid sequence comparisons of FV PR-RT with RTs from other retroviruses indicate that the polymerase domain is composed of fingers, palm, and thumb subdomains followed by a connection subdomain and the RNase H. Comparable to HIV RT, the connection subdomain appears to play a role in primer/template binding, protein stability, and polymerization efficiency. The stretch ranging from amino acid (aa) 102 to 143, which is located between the C-terminal end of the PR domain and the start of the RT domain, does not exhibit homology to retroviral PRs or any other RT, but appears to be an intrinsic part of the RT domain that is necessary for solubility and the integrity of the protein [82].

### 4.2. Polymerization Activities. 

The YXDD motif of the polymerase catalytic site is localized in the palm subdomain and is highly conserved among retroviruses. The Asp residues are involved in metal binding. A general model for the catalysis of the polymerase suggests the coordination of two Mg^2+^ ions in which one of them supports the nucleophilic attack of the 3′ OH group of the DNA primer onto the α-phosphate of the incoming dNTP, while the second metal ion is important for pyrophosphate release [83,84].

In most RTs, including the HIV-1 RT, the second site of the motif is a Met (YMDD). However, MLV and FVs contain a Val as the second residue. In HIV-1, mutation of the polymerase active site from YMDD to YVDD causes high level resistance to the inhibitor 3′ thiacytidine (3TC) [85]. Changing YVDD to YMDD in PFV severely impairs virus replication, since reverse transcription cannot be completed. In vitro polymerization assays further indicate that the wild-type YVDD PFV PR-RT is a highly processive DNA polymerase, whereas the YMDD mutant exhibits significantly reduced processivity [34], which is defined as the length of polymerization products synthesized during one round of binding and polymerization before dissociation and reassociation occur. These results indicate that FVs require a highly processive RT for efficient replication. This is probably because in contrast to HIV, only a few Pol molecules are taken up into the virus particle via the direct interaction of Pol with Gag and the viral RNA [33,34]. Interestingly, although the mutant YMDD PFV enzyme resembles the wild-type HIV-1 RT, it still is resistant to 3TC, indicating that probably additional determinants other than the Val in the YXDD motif are involved in the 3TC resistance of FVs [34].

Investigation of the fidelity of PFV PR-RT revealed that it is similar to that of HIV-1 RT for base substitutions; however, it generates more insertions and deletions [33]. Nevertheless, the genetic variation of FV genomes is limited. This might be because although FV genomes can be found in many tissues, high levels of viral RNA were only detected in oral tissues [86]. Compared to HIV, the replication activities of FVs are restricted to certain tissues, which probably supports the conservation of the genome.

Comparison of the K_M_ and k_cat_-values for polymerization on homopolymeric and heteropolymeric substrates indicated similar results for purified SFVmac and PFV PR-RT. The K_M_ values for both substrate types are also comparable [74,76]. However, the K_M_ values for FV PR-RTs are about five to 30-fold higher than published values for HIV-1 RT [87,88,89]. In addition, K_D_ values for DNA/DNA (PFV 44.4 nM; SFV 36.4 nM) or DNA/RNA (PFV 9.9 nM; SFV 32.4 nM) substrates are much higher than those determined for HIV-1 RT, for which the K_D_ values for both substrates of ca. 2 nM have been determined [90,91]. Comparison of the pre-steady state kinetics of dNTP incorporation of PFV PR-RT with HIV-1 and MLV showed a severely reduced primer extension capacity of PFV PR-RT at low dNTP concentrations. This behavior is similar to MLV RT, but in strong contrast to HIV-1 RT [92]. For example, k_pol_/K_D_ values for dATP incorporation for PFV PR-RT and MLV RT reach values of 2.9 and 2.1, respectively, whereas a value of 55.3 was achieved for HIV-1 RT [92,93]. The authors suggest that the different polymerization properties might have evolved, because HIV and FVs as well as MLV replicate in different cell types. Whereas HIV is able to efficiently propagate in non-dividing cells that have low dNTP concentrations, MLV and FVs replicate in dividing cells. Since these cells contain high dNTP concentrations, FVs did not need to evolve an RT enzyme with high dNTP binding affinities. 

## 5. RNase H Activity and Structure

The catalytic activity of the RNase H domain of retroviral RTs is essential during reverse transcription. Mutations that inactivate the RNase H prevent virus propagation [94,95]. Retroviral RNases H are partially processive endonucleases, which in general do not cleave sequence-specifically. Cleavage of the RNA strand of an RNA/DNA hybrid takes place in the presence of Mg^2+^ ions and results in 5′phosphate and 3′ OH termini. RNase H also exhibits a 3′ to 5′ exonuclease activity during DNA polymerization [96,97,98]. In addition, during reverse transcription, two specific cleavages are required to remove the extended tRNA and PPT primers, which are used to start minus-strand and plus-strand DNA synthesis. RNase H cleaves specifically between the RNA–DNA junctions [99,100,101,102,103].

To investigate cleavage at the FV PPT-U3 region, DNA/RNA substrates were designed using 5′ end-labeled RNA that contained the entire PPT and part of the U3 region of FV. During reverse transcription, FV PR-RT progressively degrades the RNA until it encounters the PPT. It was shown that FV PR-RT recognizes its own PPT, and cleaves specifically at the U3/PPT boundary. However, it did not properly cleave a similar substrate containing the HIV-1 U3–PPT junction, and vice versa HIV-1 RT did not cleave the FV substrate correctly, suggesting that the two enzymes bind the substrate differently [33]. Gel filtration and NMR data showed that the separate PFV RNase H is a monomer [77,78]. Although the presence of Mg^2+^ ions in the RNase H catalytic center is not required for substrate binding by RTs, NMR spectroscopy indicated that the metal ions are important for stabilization of the overall structure of PFV RNase H [77]. In addition, it has been shown for other RNases that RNA cleavage is achieved by a mechanism that involves two Mg^2+^ ions bound in the catalytic center [104,105].

The K_M_ values for the RNase H activity of the full-length SFVmac (18.1 nM) and PFV (17.1 nM) enzymes are similar to that of HIV-1 RT (25 nM). This is somewhat surprising, since the amount of RT molecules is much higher in HIV-1 than FV virions [33,34,74].

The isolated RNase H domain of HIV-1 RT is inactive, but activity can be restored by N-terminal extensions, which stabilize the protein [106]. Independent MoMLV and PFV RNase H retain cleavage activity; however, it is remarkably lower than that of the full-length RT enzymes [77,79,107,108]. The RNase H cleavage patterns of the independent PFV RNase H domain and the full-length PR-RT differ, and the K_D_ value of 23 µM for DNA/RNA substrate binding for the free RNase H is about 4000-fold higher, indicating a substantial role of the polymerase domain for nucleic acid affinity and specificity [74,78].

Moreover, analysis of the RNase H cleavages performed by full-length FV PR-RT and HIV-1 RT on non-specific RNA/DNA substrates revealed different cleavage sites, which also suggests differences in nucleic acid binding [33]. Time-course experiments with PFV and SFVmac PR-RTs indicate that both enzymes cleave endonucleolytically at around −17 to −19 in the RNA. This is followed by a 3′ > 5′ directed processing of the RNA [33,74]. Amino acid sequence comparisons of the RNases H from various retroviruses as well as the human and the *Escherichia coli* RNases H showed that in contrast to the inactive free HIV-1 RNase H, they all contain an additional helix-loop structure, the basic protrusion, which consists of the so-called C-helix and a downstream basic loop element [78]. The basic protrusion of RNases H has been suggested to be important for substrate binding and activity. In HIV-1 RT, this function is probably fulfilled by positively charged residues located in the connection subdomain [109,110,111].

**Figure 3:**
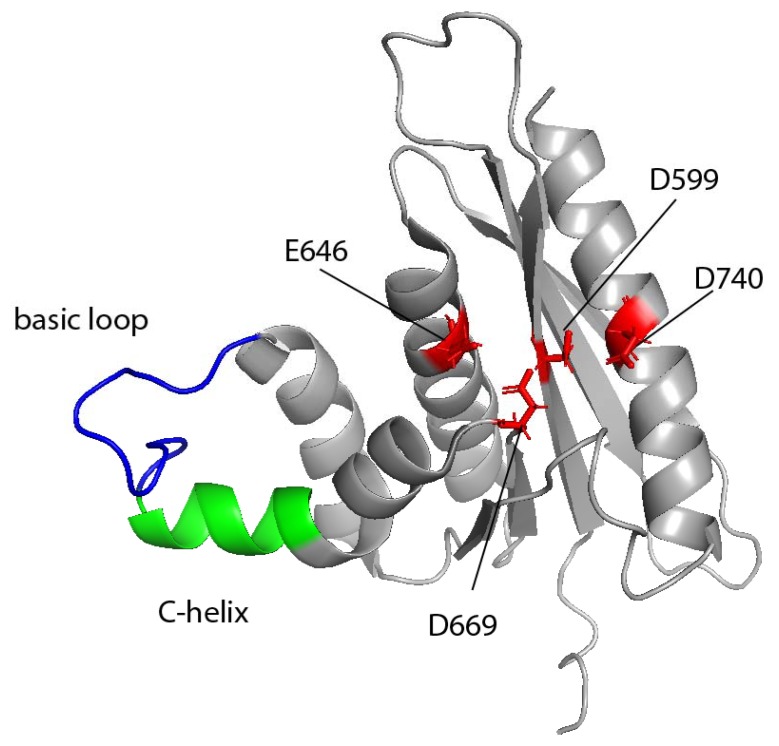
Ribbon diagram of the prototype foamy virus (PFV) RNase H structure. The C-helix is highlighted in green; the basic loop in blue. The active site residues D599, E646, D669, and D740 are depicted in red as sticks (pdb: 2LSN).

The structure of the PFV RNase H exhibits the typical fold of an RNase H, which consists of a five-stranded mixed β-sheet flanked by five α-helices (Figure 3). The PFV RNase H structure most closely resembles those of XMRV and HIV-1, even though HIV-1 lacks the basic protrusion [78,112,113]. The catalytic core consists of the highly conserved residues D599, E646, D669, and D740. Helix C precedes the basic loop, which contains four Lys (KKKPLK). On the contrary, in XMRV RNase H, the consecutive basic residues are three Arg, which are part of helix C [78,112]. The structural similarity of the HIV-1 and PFV RNase H was used to examine whether PFV RNase H can serve as a model enzyme for HIV-1 RNase H inhibitors. Indeed, several HIV-1 RNase H inhibitors were identified that also bind and inhibit PFV RNase H at low µmolar concentrations, which are similar to those of the HIV-1 RNase H. Based on NMR binding experiments with PFV, RNase H and the HIV-1 RNase H inhibitor RDS1643 structural overlays with both enzymes, and in silico docking experiments were performed to propose the inhibitor binding site in HIV-1 RNase H [114].

NMR titration experiments were performed to identify the residues involved in RNA/DNA substrate binding. ^1^H-^15^N heteronuclear single quantum coherence (HSQC) spectra of purified ^15^N-labeled PFV RNase H were recorded after the addition of increasing amounts of substrate. Chemical shift changes indicated that apart from the active site residues, residues in helix B, helix C, and the basic loop participate in binding of the substrate (Figure 3). The orientation of helix C is established by several hydrophobic contacts with helix D. This interaction enables helix C to correctly position the basic loop toward the nucleic acid substrate. Only then can proper RNA cleavage—and, if necessary, specific cleavage—be guaranteed [78].

## 6. Protease Activity and Structure

The PR activity of FVs is essential for virus production. When processed Gag in combination with a PR-deficient Pol was provided during virus production, infectious virus particles containing viral DNA were obtained, indicating that PR activity is not absolutely required at cell entry. However, infectivity was reduced to 0.5% to 2% of the wild-type infectivity [23]. Thus, other groups suggest that Gag cleavage is essential for viral infectivity [19,20]. However, PR-mediated Gag processing is absolutely necessary to initiate intraparticle reverse transcription as well as the template switch of reverse transcriptase [23,26]. What is more, Pol processing is essential for genome integration, but not for the RT activity itself [23,72].

**Figure 4:**
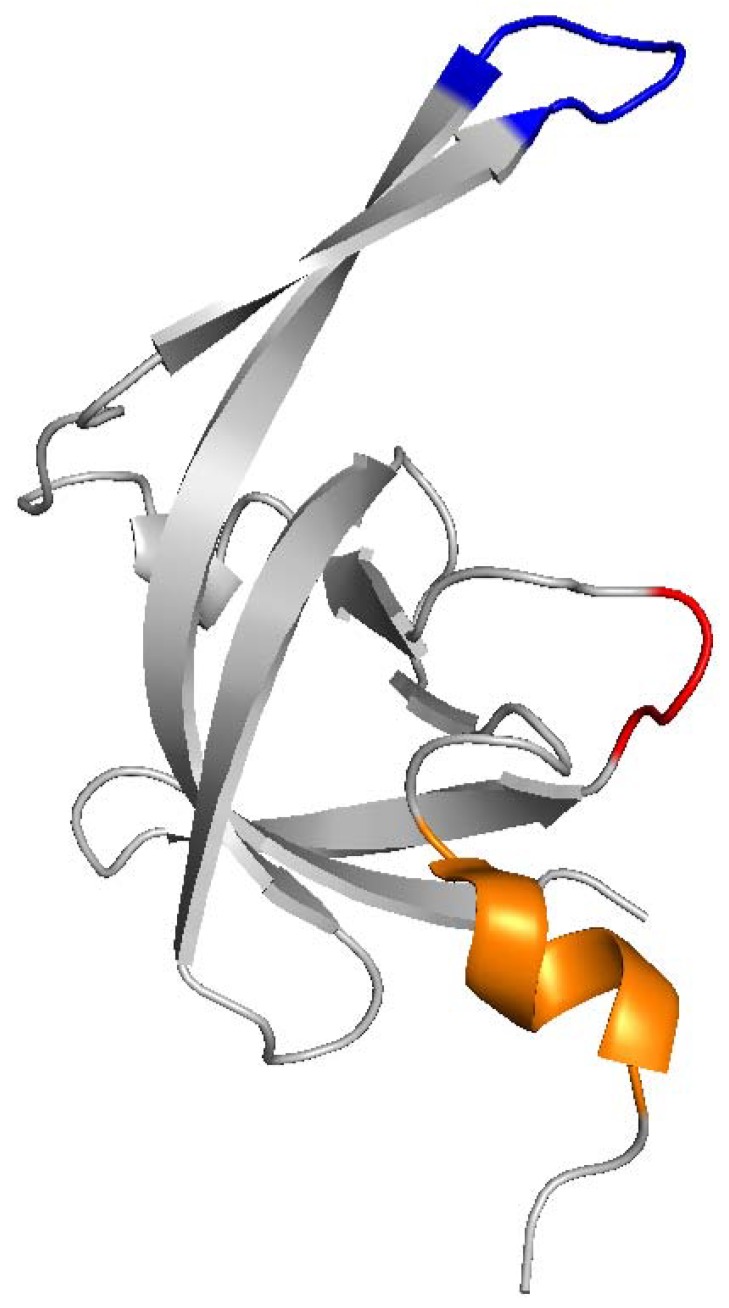
Three-dimensional structure of the SFV from macaques (SFVmac) protease (PR) monomer. The flap region (blue), the a-helix (orange) and the location of the DSG motif (red) forming the active site in the dimer are highlighted (pdb: 2JYS).

Since retroviral PRs have been shown to be only active as dimers and FV PR-RTs are monomeric proteins, the question arises how the activation of PR can be achieved [74,80,115]. The NMR solution structure of the independent SFVmac PR domain (residues 1 to 102) showed that it is a stable monomer and adopts a conformation similar to one subunit of the HIV-1 PR dimer [75,116] (Figure 4). The monomer consists of seven β-strands and a helical turn. The β-strands form a closed barrel-like β-sheet. A β-hairpin is formed by the amino-terminal halves of β4 and β5, which is typical for the so-called flap region of aspartate PRs [75,117]. Similarly to other retroviral PRs, the FV PR domain harbors four characteristic structural features: (a) a hairpin containing the A1 loop, (b) the B loop or the so-called fireman’s grip, which includes the conserved amino acid motif DSG (in some PRs DTG) forming the active site in the dimer, (c) an α-helix, and (d) the flap region [75]. Structural analyses of other retroviral PRs revealed that the fireman’s grip, the flap, the N-terminal region, and the C-terminal region, which form a four-stranded β-sheet, are involved in dimerization [117], corroborating that the FV PR domain is also able to form dimers.

Nevertheless, activity of the independent PR domain (1 to 102) as well as of the full-length FV PR-RT could only be achieved using high NaCl concentrations of 2 to 3 M [74,80,118]. The expression of PFV PR as a maltose-binding protein (MBP) or thioredoxin fusion at the N-terminus as well as a C-terminal extension of the PR (residues 1 to 143) appeared to improve the stability of the PR and allowed substrate cleavage, but activity was lost after elimination of the fusion protein [73,118,119]. Based on sequence alignments with HIV-1 PR, single (Q8R, H22L, S25T, T28D) and double (Q8R-T28D, H22L-T28D) mutants of PFV PR were created that harbored amino acid exchanges, making the PR variants more similar to HIV-1 PR. Urea denaturation revealed an increased stability for most mutants, suggesting that the substitutions promote dimer stability [120].

The putative PR dimerization inhibitor cholic acid inhibited the activity HIV-1 and FV PR, whereas darunavir and tipranavir—which are known to prevent HIV-1 PR dimerization—had no effect on FV PR. Determination of the binding site for cholic acid by ^1^H-^15^N HSQC experiments using ^15^N labeled PR indicated that the inhibitor binds in the putative dimerization interface. Paramagnetic relaxation enhancement (PRE), an NMR method that allows the detection of minor conformational species, finally showed that the FV PR domain is able to form transient homodimers. However, in solution, these dimers constitute only a small fraction of less than 5% [80,81].

Obviously, high NaCl concentrations do not represent the situation in a living cell in which the virus replicates. PR activation of HIV-1 is achieved by the formation of transient PR dimers in the Gag–Pol precursor, which leads to N-terminal autoprocessing [121]. Since FVs express Gag and Pol separately, and Pol can only be taken up into the virus particle by binding to the pregenomic viral RNA, it isobvious that FVs developed a different mechanism for PR activation. A PR-activating RNA motif (PARM) was identified in the cis-acting CasII sequence of the RNA, which includes the A and B elements of the purine-rich sequences located at the 3′ end of *pol* (Figure 1) [122].

**Figure 5:**
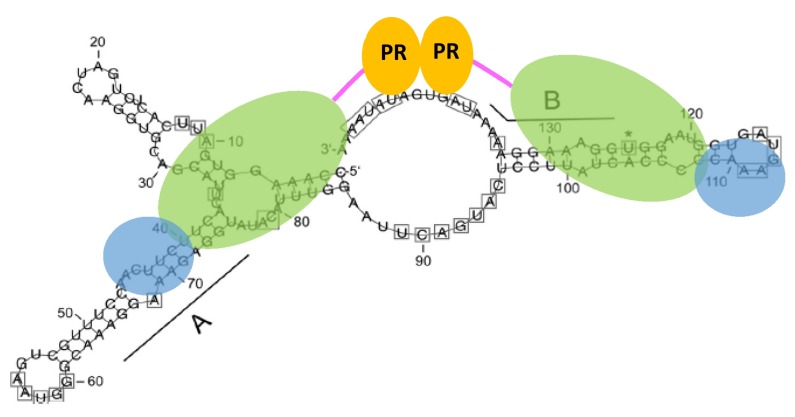
Model of protease (PR) activation upon binding to the PR-activating RNA motif (PARM). Both the A and B elements are required. They are involved in stem structures to which two PR-RT molecules can bind. Upon interaction of the RT domain with the RNA, the PR domains of the two PR-RTs can dimerize. Colors as in Figure 2.

The addition of PARM RNA to PFV PR-RT initiates substrate cleavage. The corresponding DNA does not lead to PR activation. Truncated PARM RNA or the addition of only the A or B element RNA to the assay also resulted in a loss of PR activation. Gel shift experiments with the PARM RNA and PFV PR-RT showed that the enzyme oligomerizes upon RNA binding [122]. Determination of the PARM RNA secondary structure using selective 2′ hydroxyl acylation analysed by primer extension (SHAPE) revealed that both the A and B elements are located in a stem-loop structure of ca. 15 nucleotides in length. PARM enables the formation of proteolytically active PR-RT dimers (Figure 5). It might also be possible that only the PR domains of two full-length enzyme molecules dimerize upon PARM binding [122]. The data suggest that in the host cell, the PR domain in the Pol precursor is inactive until enough viral RNA is produced. PR activation can only be achieved during packaging upon binding of the RT domain to the PARM of the pregenomic RNA. The IN domain of Pol is not required for PR activation [55]. This order of events creates a regulatory mechanism by which premature Pol or Gag processing can be avoided.

## 7. Resistance of FV PR-RT against RT Inhibitors

The only known RT inhibitors that impair PFV replication are tenofovir and azido-3′-deoxythymidine (AZT, zidovudine). The addition of 5 µM of AZT to cell cultures are sufficient to prevent virus propagation [45,123,124]. Attempts to generate AZT resistant FV were only successful with SFVmac, but not with SFV from chimpanzee (SFVcpz) or PFV. This is quite astonishing, since the amino acid sequences of the polymerase domains of PFV and SFVmac are 84.5% identical.

**Figure 6:**
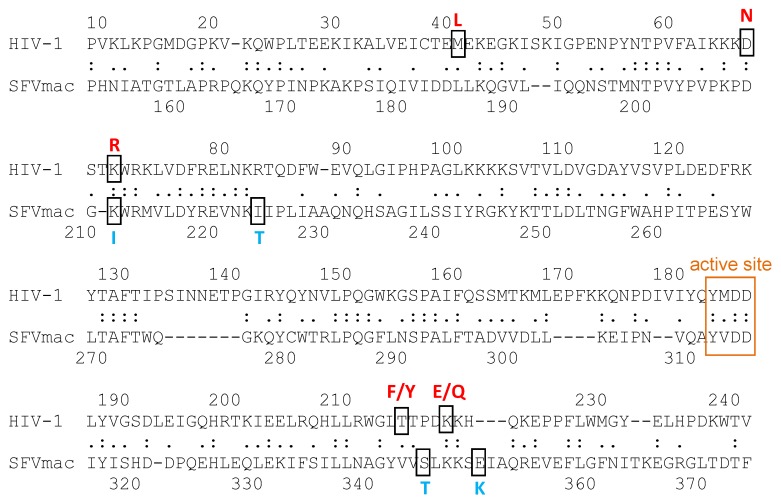
Sequence alignment of the regions of human immunodeficiency virus (HIV)-1 and SFVmac RT, showing the azido-3′deoxythymidine (AZT) resistance amino acid exchanges. The amino acids conferring AZT resistance are shown in red for HIV-1 (M41L, D67N, K70R, T215Y/F, K219E/Q) and blue for SFVmac RT (K211I, I224T, S345T, E350K), respectively. The amino acids of the polymerase active site are highlighted by an orange box. The amino acid identity is 26.2%.

Four amino acid substitutions in the RT domain of SFVmac have been identified that together confer high-level resistance to AZT: K211I, I224T, S345T, and E350K (Figure 6). The I224T substitution is probably a polymorphism that does not contribute directly to AZT resistance, but is important for regaining polymerization activity and viral fitness [76,125]. Two different AZT resistance mechanisms have been shown for HIV: HIV-2 is able to discriminate between the natural triphosphate TTP and the phosphorylated inhibitor AZTTP, whereas the major mechanism in HIV-1 is based on the removal of the already incorporated chain-terminating AZTMP in the presence of ATP [126]. In SFVmac, the AZT-resistant RTs can also remove the incorporated AZTMP more readily than the wild-type enzyme in the presence of ATP. The PR-RT harboring the single amino acid exchange S345T is the only single substitution variant exhibiting significant AZTMP excision activity. Excision efficiency doubles when K211I is present together with S345T or E350K [127].

In AZT-resistant HIV-1 RT, the aromatic amino acid exchange T215F/Y allows π–π stacking interactions with the adenine ring of ATP and thus more efficient AZTMP excision [128,129]. In AZT-resistant SFVmac RT, instead of acquiring an aromatic residue, the most important substitution is S345T. NMR ^1^H-^15^N HSQC experiments with truncated wild-type and resistant SFVmac RTs harboring the fingers and palm subdomains of the polymerase were recorded in the absence and presence of ATP. Comparison of the spectra revealed that a Trp residue is involved in ATP binding in the S345T variant, which is obscured in the wild-type enzyme, suggesting a direct contact of ATP via π–π stacking interactions similar to HIV-1 RT.

## 8. Outlook and Persepectives

The life cycle of FVs differs from that of conventional retroviruses in various aspects. Several of the molecular details have been elucidated that make us aware of the differences that have developed during the evolution of FVs. The structure of some FV proteins is already known: PR, RNase H, and IN, as well as parts of the Gag protein [75,78,130,131,132,133,134]. The structure of the full-length FV PR-RT is still missing, but would contribute greatly to our understanding of RTs in general. So far, the only monomeric RT 3D structures known are those of XMRV RT and the closely related MLV RT [70,71]. In addition, the crystal structure of the yeast retrotransposon Ty3 RT has been solved, which is a monomer in solution, but dimerizes upon substrate binding [135]. In order to fully understand the function and mechanistic details of FV proteins and enzymes, more structural and functional information is urgently needed.
